# Evaluating the Effects of Disinfectants on Bacterial Biofilms Using a Microfluidics Flow Cell and Time-Lapse Fluorescence Microscopy

**DOI:** 10.3390/microorganisms8111837

**Published:** 2020-11-22

**Authors:** Milos Legner, James Jonkman, Dean Swift

**Affiliations:** 1 Micrylium Laboratories, Toronto, ON M3H 5T5, Canada; swift@micrylium.com; 2 Advanced Optical Microscopy Facility, University Health Network, Toronto, ON M5G 1L7, Canada; james.jonkman@uhnresearch.ca

**Keywords:** biofilms, disinfectants, microfluidics, fluorescence microscopy, time-lapse imaging

## Abstract

A commercially available microfluidics flow cell was utilized together with widefield fluorescence microscopy to evaluate the effects of disinfectants on bacterial strains. The flow cell’s inner surface supports the formation of biofilms of numerous bacterial species. The modular setup of the flow cell accessories allows connection to syringes, pumps and collection vials, facilitating aseptic experiments in a controlled fluidics environment which can be documented with precisely timed microscopy imaging. The flow cell is inoculated with a suspension of bacteria in a nutrient medium and incubated for several days allowing bacterial cells to form a biofilm. Shortly before performing an assay, the biofilm is labelled with a dual-fluorescent DNA probe which distinguishes unharmed and damaged bacteria. Then a disinfectant sample (or control) is gently injected and time-lapse imaging is used for quantifying the course of bacterial biomass response. We use a simplified widefield microscopy method that allows intensive recording and quantification of time series of two-dimensional frames for tracking the course of disinfectant action on a variety of microbial strains. This procedure has potential for the rapid evaluation of novel products.

## 1. Introduction

Although the capability of biofilm formation has been recognized as a powerful trait of many microbial species for decades [1], the striking increase of tolerance against antimicrobial agents as a result of switching from planktonic to biofilm phenotype has sounded an alarm only recently [2].

The disinfectant industry has gradually accepted the idea of using biofilm-based methods such as the Minimum Biofilm-Eliminating Concentration (MBEC) assay [3] for testing new products; however, sufficient attention has not been paid to the time course of the biofilm breakdown and the impact of agents on various types of biofilm organisms. Procedures combining time-lapse capability with visualization of cellular-level changes, when standardized, would give us a better tool for choosing among the ever-growing number of antibiofilm technologies.

Previous pilot experiments suggested that using nucleic-acid fluorescent probes helps visualize the dynamics of disinfectant action in bacterial biofilms [4,5]. In a more detailed study on hydroxyapatite discs using confocal laser scanning microscopy (CLSM), Xiao et al., 2012 [6] were able to quantify the impact of one agent in several consecutive 15-min intervals using specialized software. More recently a “bulk” approach using a microplate reader [7] cast more light on the dynamics of fluorescent probes in live and dead bacterial cells.

Our goal was to develop a quantitative procedure bridging the gap between bulk methods and detailed (confocal) microscopy studies. To this end, flow cells appeared to be the first choice apparatus, as they were considered a favourite tool for biofilm research. However, the most frequently used microfluidics system (BioFlux, e.g., [8,9]) was not suitable for our purpose without major adaptations. Moreover, the width of its channels is about half of the intended width of the microscope field and a pre-requisite for seeding inner surfaces with bacteria is priming with a “pellicle” forming fluid, e.g., spun saliva. Consequently, we chose the Ibidi Flow Cell (Slide VI^0.4^ Ibi Treat; Ibidi, Fitchburg, WI, USA) with approximately a 60-fold larger channel profile which diminishes the negative effects of viscous forces and generation of micro-bubbles.

As a starting point, we decided to use (1) a low-power, high Numerical Aperture (NA) objective to assess large (ca 0.4 mm^2^) areas of biofilm that captures the emission of the entire thickness of the biofilm, (2) a high-sensitivity sCMOS camera that requires a fraction of the illumination energy normally needed for the CLSM (confocal) system and (3), the open source software package (FIJI ImageJ by the National Institutes of Health, USA) to binarize the entire time series of frames simultaneously. We also acknowledge that a substantial drawback of our initial experiments using time series of microscopic images [4,5] was the delivery (injection) of agents in a manual fashion that prevented standardizing the procedure and did not allow us to pinpoint the moment when the leading edge of injected fluid (disinfectant agent) arrives in the viewing area. The introduction here of a precise Linear Pump solves those issues.

## 2. Materials and Methods

### 2.1. Bacterial Strains

To develop the method, we chose two Risk Group 1 strains from the ATCC collection: *Staphylococcus epidermidis* (Winslow and Winslow (Evans); ATCC 12228), a non-motile, non-pathogenic, Gram-positive coccus (typical component of a normal human skin microbiome) and a strain of Gram-negative *Escherichia coli* (ATCC 8739). We also tested the former species with an over-the-counter available mixed culture of probiotic strains (Ultimate Flora Critical Care; Lot 1816701; Renew Life Canada, Brampton, ON, Canada).

### 2.2. Disinfecting Agents

For initial screening of the novel method potential we used several commercial agents (not identified by name here) based on similar components:

Product **“A”** is a wipe disinfectant. For the purpose of these assays, it was extracted (and diluted) from disinfectant impregnated wipes (DIW; rectangular cloth 7.5” × 9.0” soaked with the disinfectant) back into deionized (DI) water as follows: Approximately 0.8-inch (2 cm) strips were cut out with scissors, rolled up (using gloves) and put in pre-weighed 15-mL (“Falcon” centrifuge) tubes. The weight of the wipe tissue was calculated from the increment (in grams). Then 7× greater amount of DI-water (in mL) was added to each tube so that the tissue represented about ⅛ of the total weight. The tissue was then packed (squeezed) into the tip of the tube with a pipette, the tube was vortexed and a 2.5-mL aliquot of liquid was transferred into a new clean tube (any other amount can be chosen that can be withdrawn from all tubes). After proceeding with each sample, the tubes were spun for 20 min at 3000 g in a bench-top centrifuge. After spinning, the supernatant fluid (ca 2 mL each) was decanted to new clean tubes (final samples to be used for assays). This product was tested in 2 assays (8 replicates in total).

We call Product **“B**” the original liquid used for soaking the DIW. It contains 19.9% ethanol (*w*/*w*) and 0.1% chlorhexidine gluconate (*w*/*w*) dissolved in DI water. It also contains non-ionic surfactants and essential oils. This product was tested in 3 assays (7 replicates in total).

Product **“C”** is a liquid disinfectant containing 9.5% ethanol (*w*/*w*) and 0.12% chlorhexidine gluconate (*w*/*w*) dissolved in DI water. It also contains viscosity control agents. This product was tested in 3 assays (5 replicates in total).

Product **“D”** contains 70.5% ethanol (*w*/*w*) and 0.2% chlorhexidine gluconate (*w*/*w*) dissolved in DI water. It also contains surfactants and polyols. This product was tested in one assay (3 replicates in total).

### 2.3. Widefield Microscopy

For the acquisition of time series that formed the core of our assays, we used a Zeiss AxioObserver inverted widefield microscope (Carl Zeiss, Jena, Germany) equipped with a monochrome Hamamatsu Flash 4 (sCMOS 2048 × 2048 pixel) camera (Hamamatsu Photonics, Japan), which has a maximum full resolution frame rate of 100 fps (frames/sec). We used a 20×/0.8NA Plan-Apochromat objective lens that allowed us to obtain square frames 666 μm wide. Using the XCite 120Q fluorescence lamp (Excelitas, Mississauga, Canada), green fluorescence was excited with a 450–490 nm bandpass filter and captured with a 500–550 nm bandpass filter; red fluorescence was excited with 540–580 nm band and captured at 595–665 nm. The digital arrays are displayed either in a pseudo-colour of the respective band or as a composite image (green and red superimposed).

### 2.4. Confocal Microscopy

For detailed three-dimensional imaging of the used bacterial strains in the channels of the Ibidi flow cell (see 3. Recommended Procedure) we employed confocal laser scanning microscopy (CLSM) using a Zeiss LSM700 (Carl Zeiss, Jena, Germany) with a 63×/1.4NA oil immersion lens (maximum resolution is about 0.2 µm laterally and about 0.5 µm in depth). For this study, we captured square frames 101.6 μm wide. Green fluorescence (490–530 nm) was excited with a laser wavelength of 488 nm while the red fluorescence (600–700 nm) was excited with the 555 nm line.

### 2.5. Live/Dead Fluorescence Labelling

After being exposed to fluorescent probes Syto 9 and propidium iodide (PI) that intercalate in the chromosomal DNA, bacterial cells become double labelled. Syto 9 causes all cells, live or dead, to emit green fluorescence, whereas the red (PI) fluorescence stays weak unless the plasma membrane of a cell becomes permeable, indicating that the cell has been lethally damaged [10]. Typically, a bacterial population contains a fraction of dead or dying cells. In cell imaging that uses superposition of green and red pixels, some resulting composite images of cells or their clusters become red while some appear yellow, co-localizing relatively strong emission of both green and red signal. Both types of cells are regarded as non-viable [11].

### 2.6. Biofilm Formation

**Figure 1:**
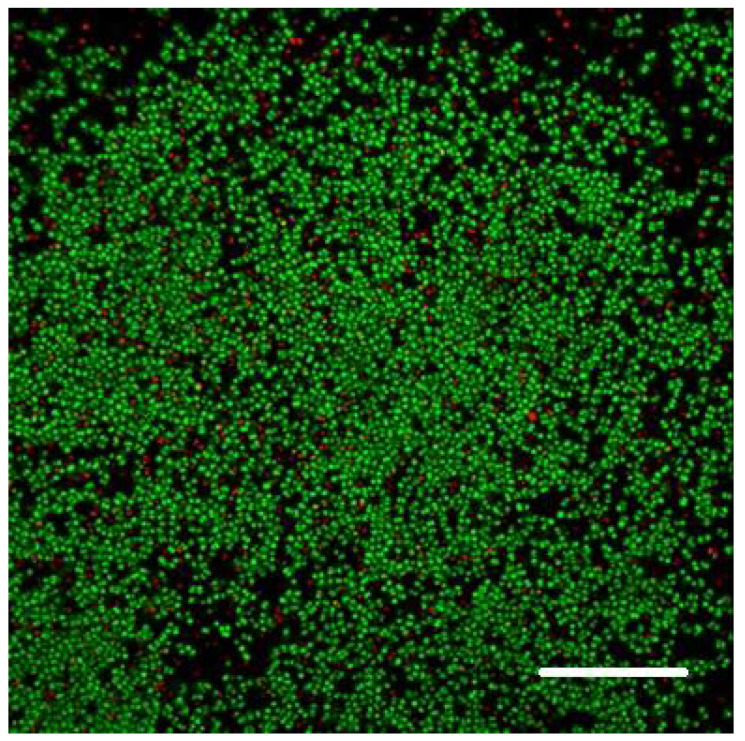
Cells of *S. epidermidis* forming a layer on the inner surface of the Ibidi flow cell channel. Single frame (slice) of a confocal stack. Composite image of green and red emission bands: green fluorescence (Syto 9) was excited with 488 nm laser; red fluorescence propidium iodide (PI) was excited with 555 nm laser; scale bar = 20 µm.

**Figure 2:**
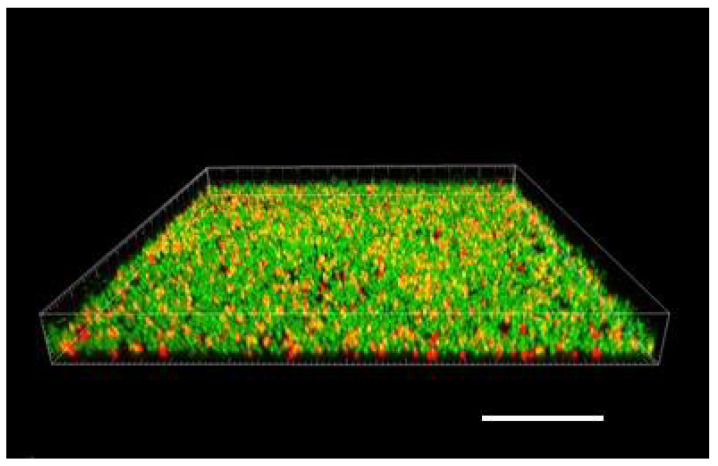
Three-dimensional rendering of a biofilm layer of *S. epidermidis* cells created from a confocal stack using Imaris software (Bitplane United States, Concord, MA). Side of the square frame 101.6 µm; height (z-dimension = focus range) 9.4 µm; scale bar applies to the front lower edge = 20 µm. Composite image of two emission bands (same as Figure 1).

**Figure 3:**
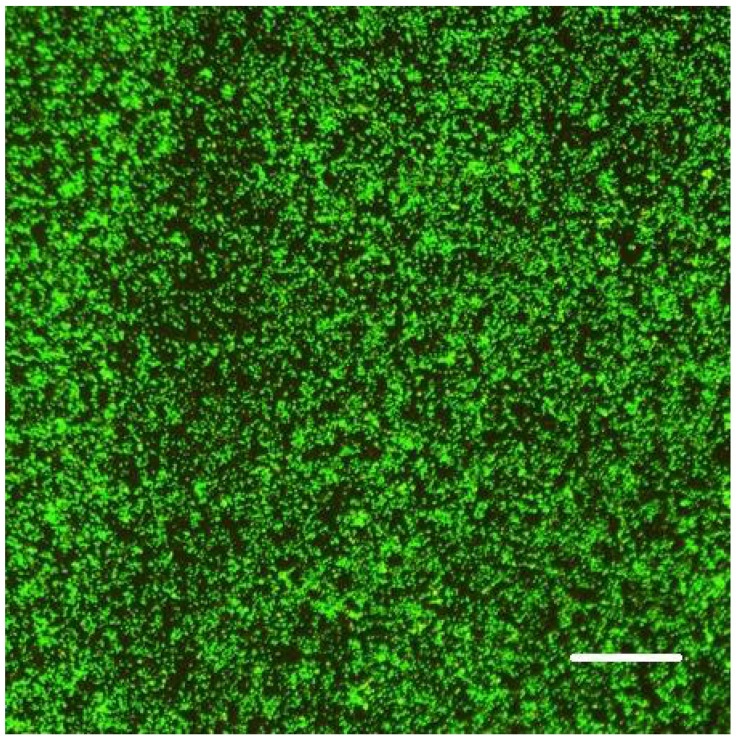
Widefield microscopy view of the *S. epidermidis* biofilm in the Ibidi flow cell channel. Composite image of green (G) and red (R) fluorescence; exposure G = 80 ms; R = 100 ms) with 20×/0.8NA lens of AxioObserver microscope (scale bar = 100 µm).

We allowed bacteria to form biofilms on the inner surface of channels of the Ibidi flow cell for at least 48 h (after injecting their culture together with fresh nutrient medium typically supplemented with 0.5% or 1% sucrose; see 3. Recommended Procedure; Figure 1, Figure 2 and Figure 3).

When raised in monoculture in our flow-cell assembly, *S. epidermidis* formed a tightly packed biofilm, as demonstrated by confocal images (Figure 1 and Figure 2). While the confocal stacks were informative about the biofilm architecture and the optical sectioning provided high contrast images, for our quantitative time series, we used lower magnification widefield snapshots that provided a substantially larger field of view, a depth of focus that includes the entire biofilm, and took much less time to acquire (on the order of 0.1 sec per each red or green channel per frame, as opposed to minutes for the 3-D confocal z-stack; Figure 3).

**Figure 4:**
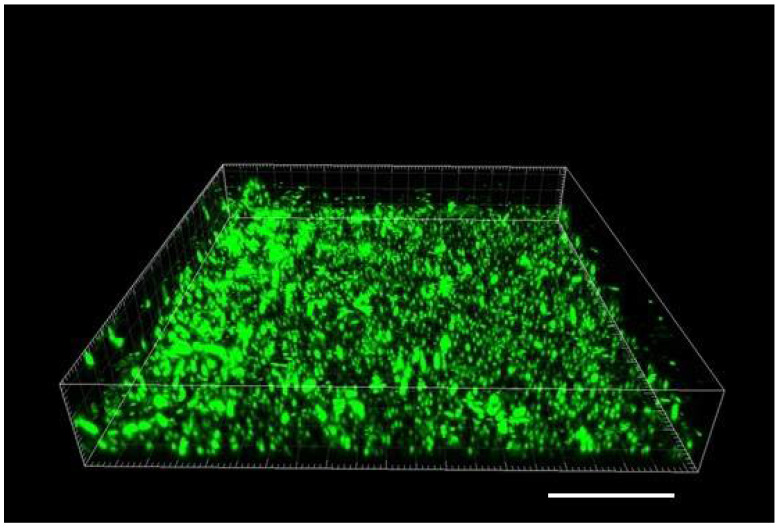
Three-dimensional rendering of a group of *E. coli* cells located on the top of biofilm in a channel of Ibidi cell. Side of the square frame 101.6 µm; height (z-dimension) 17.0 µm; scale bar applies to the front lower edge = 20 µm. Only the data of green fluorescence band (Syto 9; excitation 488 nm) were used to allow better resolution of *E. coli* rod-shaped cells. These cells appear larger than *S. epidermidis* cocci in the biofilm due to their higher brightness and slight shift during scan.

In order to attain greater diversity of the material, we initially tested a mixed biofilm of Gram-positive *S. epidermidis* and commercial probiotic culture, and then we grew a Gram-negative *Escherichia coli*. As opposed to *S. epidermidis*, the *E. coli* did not establish a biofilm, just a loosely floating culture of planktonic cells. The floating culture did not allow us to capture a good static confocal image, since the positions of individual cells were shifting during the confocal scan due to Brownian motion and partly as a result of the bacterial cells’ own motility. *S. epidermidis* did not stimulate attachment of *E. coli* when the culture was added to its biofilm with the boost medium (see 3. Recommended Procedure). Yet, in those assays, using 3-D rendering and maximum intensity projection (MIP), we found numerous rod-shaped cells of *E. coli* positioned on the top of *S. epidermidis* biofilm (Figure 4).

### 2.7. Agent Delivery

Using a Linear Pump for precise metering of the inflow into individual channels, we examined two options of agent injection:

- Slow continuous flow while simultaneously recording the time series. The time-lapse record started when the Linear Pump began metering the fluid from a syringe.
- Precise timing of the agent delivery that would displace the fluid layer above the biofilm and stop the flow. The time-series recording would start immediately after the flow had stopped.

**Figure 5:**
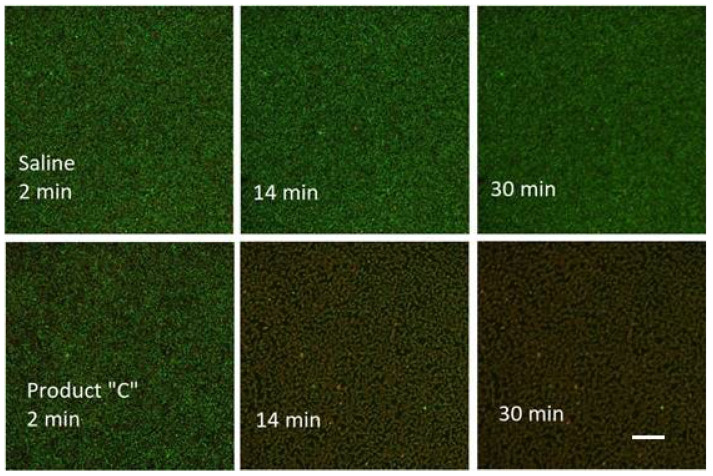
Points of a time series taken with a mixture of *S. epidermidis* and probiotic culture. Snapshots (composite images of green (G) and red (R) fluorescence; exposure G = 80 ms; R = 200 ms) with 20x/0.8NA lens of Zeiss AxioObserver microscope; scale bar in the Product “C” 30 min frame applies to all frames = 100 µm).

An example of the former alternative (a) was an assay using a mixture of *S. epidermidis* and probiotic culture where the time series was recorded over a period of one hour (Figure 5). In the control run, a 2-mL dose of 0.89% NaCl was injected into the channel of the Ibidi flow cell. The same slow injection speed was used for the delivery of Product “C” (see 4. Results) in a separate run. While the appearance of the control remained constant during first 30 min, the run with the tested agent displayed an apparent change in fluorescence colour as early as in 14 min.

**Figure 6:**
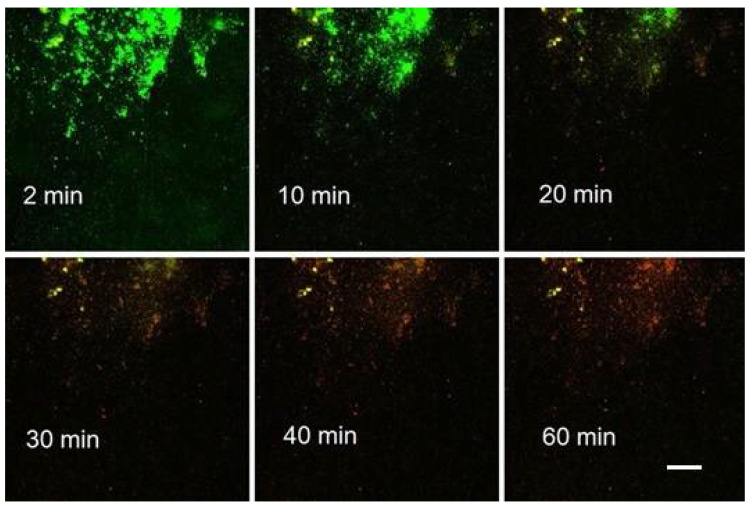
Points of a time series taken with a mixture of *S. epidermidis* and *E. coli.* Minutes are the time after bringing the leading edge of Product “A” over the biofilm. Snapshot obtained with 20x/0.8NA lens of AxioObserver microscope (composite images of green and red fluorescence; exposure of both emission channels 80 ms; scale bar in the 60 min frame applies to all frames = 100 µm).

We applied the second option (b) of the timing for pump and image recording to a mixed population of *S. epidermidis* and *E. coli* (Figure 6). Despite this combination of biofilm with loosely floating planktonic cells, precise timing optimized the agent (Product “A”) delivery. It virtually eliminated the interference of fluorescence signal by particles moving in the fluid stream, such as bacterial cells and micro-colonies. As a result, we obtained a series of virtually static images with continuously changing fluorescence colour from mostly green (emission of Syto 9) to prevailing red (emission of PI; Figure 6).

## 3. Recommended Procedure

### 3.1. Ibidi Cell Assembly

A BioSafety Cabinet (BSC, a laminar flow hood) is loaded with parts of the testing equipment. Autoclaved pre-assembled pieces of silicone tubing attached on one end to Luer-lock elbows (Elbow Luer Connector, Ibidi, Fitchburg, WI, USA; 12 pieces) are essential components. Their opposite ends are fitted with blunt veterinary needles with Luer-lock attachments (SAI Infusion Technologies, Lake Villa, IL, USA; 6 pieces for inflows) and with cut pieces of blunt needles without the Luer-lock attachments (6 pieces for outflows). Further, 3-mL disposable syringes (6), disposable hypodermic needles (7; 18G × 1”), Becton–Dickinson Falcon 5 mL Polystyrene Tubes (BD-tubes) with caps (6), an ethanol burner, forceps, a wrapped Ibidi cell (Slide VI^0.4^ Ibi Treat; Ibidi, Fitchburg, WI, USA), and a 15-cm disposable petri dish are placed into the BSC.

The BSC is decontaminated with UV light while running the air circulation (30 min). Then the caps of BD-tubes (waste collectors) are perforated with a hot hypodermic needle (using forceps) and the blunt-needle ends of silicone tubing (6) are inserted into the orifices in caps. The Ibidi cell is unwrapped from its sterile packaging and positioned on a 15-cm petri dish. Elbow-ends of inflow tubing parts (6) are plugged into one side of the cell while the elbows of outflow tubing (6) are plugged into its opposite side.

### 3.2. Inoculation

The startup culture is diluted 1:9 with fresh nutrient medium (in this study, the assays with Products “A, B” used ¼ BHI medium with 1% sucrose and the assays with Products “C, D” used ⅛ TSB medium with 0.5% sucrose). For each of 6 channels of the Ibidi flow cell, the culture is aspirated into a 3-mL disposable syringe, which is then mounted on the Luer-lock end of inflow tubing. The culture is slowly injected into each channel until it overflows to the waste collector. The inflow and outflow tubes of all channels are closed with clamps and the waste collectors are replaced with empty ones. The assembly on a petri dish is placed into a 37 °C incubator until the next treatment.

### 3.3. Replenishing Medium

Typically, fresh sterile nutrient medium is added into the channels after 2-day incubation. The BSC is decontaminated with UV light while running the air circulation (30 min). Then the assembly is brought into the BSC and the tubing is unclamped. Using 3-mL disposable syringes mounted on the inflow Luer-lock ends, each channel is slowly injected with the fresh medium until it overflows to the waste collector.

Then the inflow and outflow tubes of all channels are clamped and the waste collectors are replaced with empty ones. The assembly on the petri dish is returned to a 37 °C incubator for further biofilm growth.

### 3.4. Application of DNA Probes

**Figure 7:**
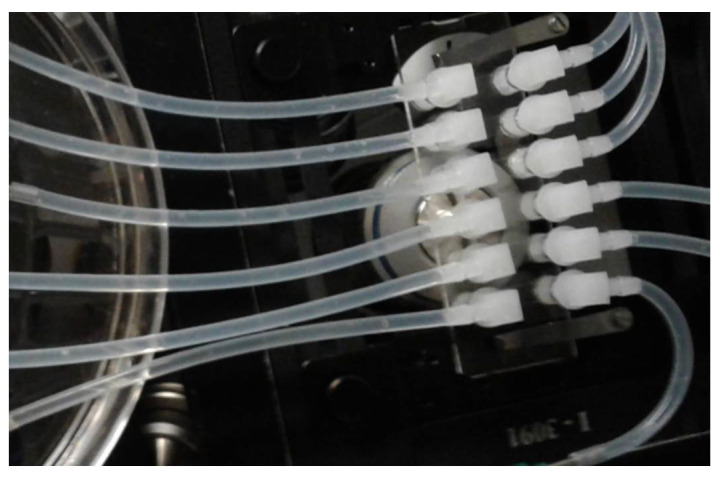
The Ibidi flow cell (Slide VI^0.4^ Ibi Treat; Ibidi, Fitchburg, WI, USA) assembly on the stage of inverted Zeiss AxioObserver microscope. Row of six elbow plug connectors attached to silicone tubing (inflow; on the right) bring fluids to 6 flow cell’s channels (not visible; 0.4 mm high, 3.8 mm wide) leading to six elbow connectors of the outflow (silicone tubing on the left). Inverted objective lens is visible under the flow cell.

After a 3-day incubation at 37 °C, the assembly is placed on a (removable) stage of a microscope (Universal Insert 160 × 110 mm, Applied Scientific Instruments, Eugene, OR, USA; Supplementary Figure S1). Clamps on silicone tubing are released. Using 3-mL disposable syringes mounted on the inflow Luer-lock ends, each channel is slowly injected with BacLight Live/Dead probe (L7012; ThermoFisher Scientific, Waltham, MA, USA, [10]) until it overflows to the waste collector. The solution is a mixture of 1.5 μL Syto 9 and 1.5 μL of propidium iodide (PI) from the manufacturer’s kit per 1 mL of 0.89% NaCl. Then the inflow and outflow tubes of all channels are clamped and waste collectors are replaced with empty ones. The Universal Insert with the Ibidi cell assembly is left for 30 min at room temperature in the dark before it is transferred to a microscope (Figure 7).

### 3.5. Linear Pump Timing

**Figure 8:**
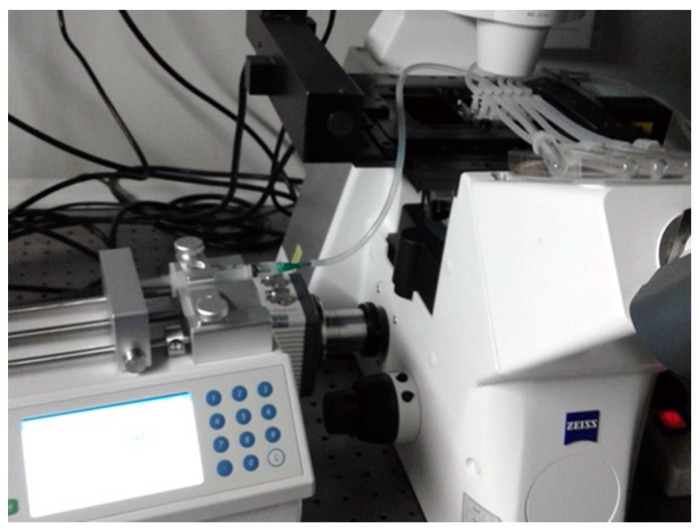
Linear pump (Fusion 100 Infusion Pump; Chemyx, Inc., Stafford, TX, USA) for accurate constant delivery of injected material (liquid disinfectant) with inserted disposable syringe connected to the flow cell on the stage of Zeiss Axiomat microscope through silicone tubing.

In order to standardize the biofilm response to different agents, we needed to estimate the timing of liquid displacement in the monitored segment of the Ibidi flow cell channel at a reproducible flow velocity. A Linear Pump was employed for driving syringes which maintains an accurate steady flow in the range 0.0001 µL to 102 mL per minute (Fusion 100 Infusion Pump, Chemyx, Inc., Stafford, TX, USA; Figure 8). The approximate time of the arrival of the leading edge of the injected fluid at the microscope field was established after the length of inflow silicone tubing had been standardized (31 cm) as follows:

A suspension of planktonic *Escherichia coli* was prepared which was grown for 24 h alternatively in LB (sample A) or BHI medium (samples B–D). About 30 min before assays, the cultures were mixed with fluorescent probes (Syto 9 and PI). After a disposable 3-mL syringe had been filled with the culture, it was inserted into the holder of the Linear Pump.

**Figure 9:**
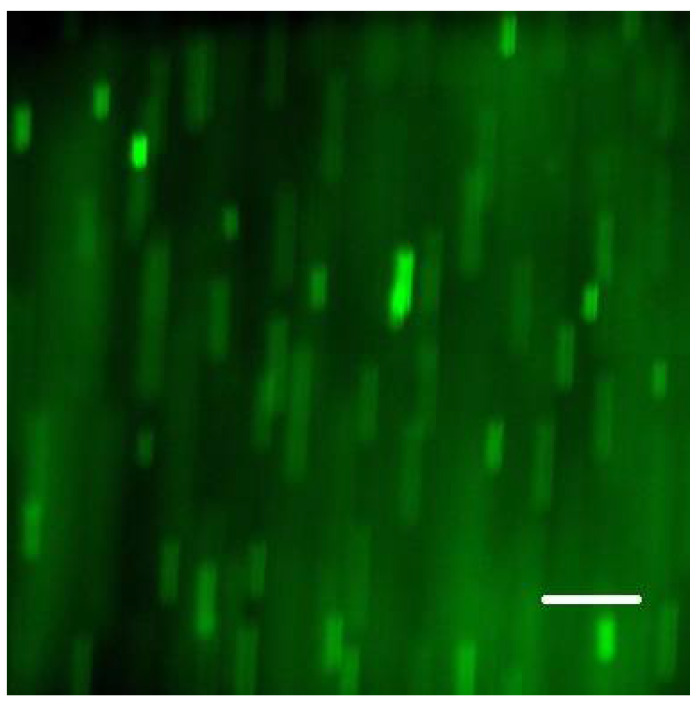
First traces of *E. coli* cells in the microscope field (55 s after start; Zeiss AxioObserver, 20×/0.8NA lens). Cells appear as smeared green spots because of long exposure time relative to the motion of cells carried by fluid (scale bar = 100 µm).

The start of pumping (at 1 mL/min) coincided with the start of time-lapse recording of microscope frames (at 5-sec intervals; see Section 3.6. Widefield microscopy time series). Post-processing of the green fluorescence (Syto 9) channel was performed using the FIJI ImageJ software (see Section 3.7. Evaluating time series). In the sequence of frames, there was an instant change from a dark field to multiple (motion-blurred) images of individual cells when the leading edge of cell suspension arrived (Figure 9). The results of the four assays are summarized in Table 1. We concluded that in the current setup, on average 1 min (or minimum 50 sec) was needed to overlay the biofilm with an agent solution.

### 3.6. Widefield Microscopy Time Series

The Universal Insert with the Ibidi cell assembly is placed on the stage of a widefield fluorescence microscope (Zeiss AxioObserver) and the silicone tubing is unclamped (Figure 7). Each time, one 3-mL disposable syringe is replaced with a new one containing a disinfecting agent. The syringe is inserted into the holder of the Linear Pump (Figure 8).

The time-lapse recording was controlled by Zeiss ZEN 3.0 (Blue Edition), which is a proprietary feature of the Zeiss AxioObserver microscope system (many other microscope software packages allow similar acquisition). The software allows programming a variable interval between timed snapshots. Individual frames (2-dimensional images) are arrays of numerical “gray values” of pixels acquired within a given wavelength band (green or red). Consistent acquisition parameters were maintained, i.e., any automation of exposure time, brightness and contrast were disabled. All post-processing was then performed with the FIJI ImageJ open-source image analysis software (see the next section).

### 3.7. Evaluating Time Series

The 2-dimensional time-lapse files obtained with Zeiss ZEN 3.0 (blue edition; typically in the proprietary format CZI) were further processed using the FIJI ImageJ software (public domain image analysis software of the National Institutes of Health, USA). To detect the first cluster of cells in the sequence of frames for the Linear Pump timing, contrast enhancement was applied to the green fluorescence (Syto 9) channel boosting the visibility on the monitor without changing the data values. For the quantification of luminance of a respective channel (green for Syto 9 and red for propidium iodide), binarizing was achieved by finding a threshold value applicable to the entire time series of frames. Typically, the default threshold found by the software for the brightest frame could be applied for this purpose.

The specific sequence of operations in FIJI was as follows:

1. Drag the image file name (.czi) to a FIJI bar (to open); when the file opens, follow the FIJI menu:
2. *Image* > *Color* > *Split channels*
3. Separately for channel C1 (green) and channel C2 (red) find the brightest frame, then:
4. *Image* > *Adjust* > *Threshold*
  - Check: Default | then Apply
  - Uncheck: Calculate threshold for each image | Check: Black background
5. *Analyze* > *Measure*
  - (take a measurement for each frame starting with frame #1 separately for channel C1 (green) and channel C2 (red)); the software generates a column (vector) table of values (Results.txt) for each channel
6. Copy the table and paste it into an Excel spreadsheet.

### 3.8. Expressing the Data as the Time of Signal Doubling or Half-Time of Signal Decay

Calculating the rate of signal change allows a more general interpretation of results. Formulas for red signal doubling and green signal decay (“halving”) are identical, derived from either the positive or negative value of the rate of change (**r**). The latter variable (in h^−1^ as a physical unit) can be calculated from the sequence of values acquired as time-lapse images (time series):

r = (ln(pix_t2_) − ln(pix_t1_))/(t2 – t1) (1)

where **pix_t1_** and **pix_t2_** are values (number of white pixels) acquired by binarizing the fluorescence images of the biofilm (using the FIJI software) and **t1** and **t2** are corresponding time points.

Then the signal doubling time or half-time (**T**; in minutes) can be calculated as:

T = (ln(2)/r) * 60(2)

## 4. Results

We did not attempt to compare, rank or otherwise evaluate the disinfectant products taken to the test. Rather, we present a set of case studies, i.e., specific examples dependent on a bacterial strain or disinfectant. Among the assays listed in the Materials and Methods, we only chose data obtained at closely comparable conditions, such as the same frame exposure time, frame recording interval and time series duration.

### 4.1. Product “A”

**Figure 10:**
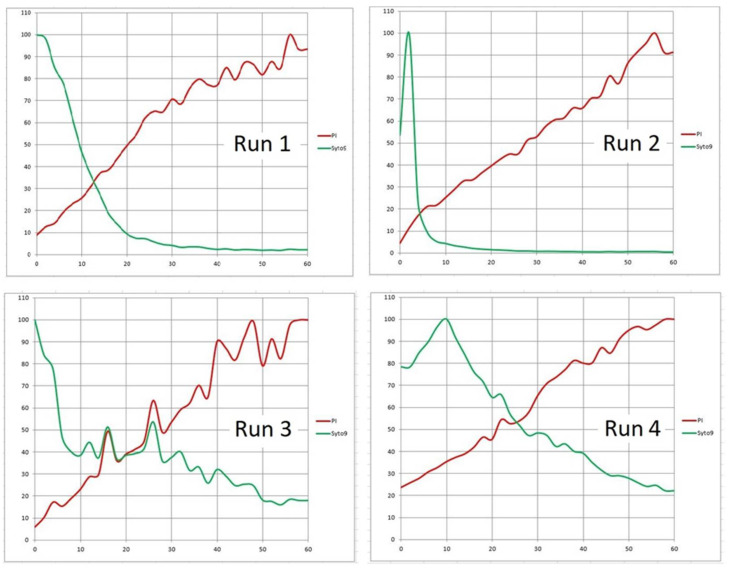
Time-lapse records of PI (red) and Syto 9 (green) fluorescence signal from a mixed biofilm *of S. epidermidis* and *E. coli* exposed to Product “A” in four separate assays. Horizontal axis: time in minutes. Vertical axis: % of maximum frame luminance.

The precise steady metering of the Product “A” and stopping the pump before the time series started (delivery option (b); see 2. Materials and Methods) was aimed at improving the reproducibility of assays by decreasing the number of variables. Using this approach, we achieved steady images even with a relatively loose biofilm consisting of *Staphylococcus epidermidis* and *Escherichia coli*. (Figure 6; see Section 2. Materials and Methods). In that particular run, the sample of Product “A” was delivered at 0.5 mL/min. Starting 2 min after the delivery had stopped, the 1-h time series (Run 1) was recorded in 2-min intervals (see 3. Recommended Procedure). After binarizing individual frames, a graph expressing the course of fluorescence emission in the percentage of the brightest frame revealed crossing lines of decreasing Syto 9 (green) and increasing PI (red) fluorescence signal (Figure 10; Run 1).

**Figure 11:**
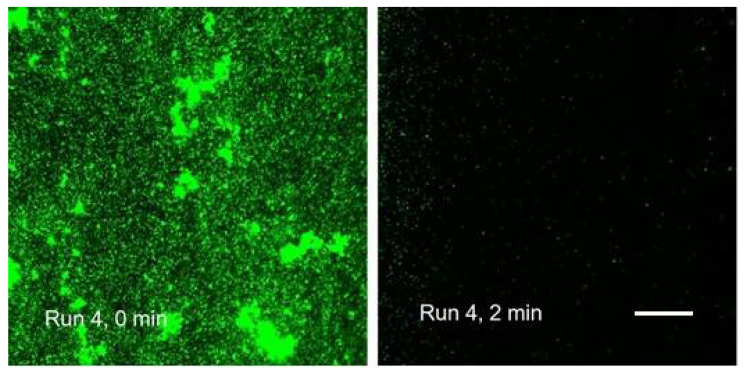
First two frames of a time-lapse record of Run 4 (20×/0.8NA lens of AxioObserver microscope; scale bar in the 2 min frame applies also to the 0 min frame = 100 µm).

In additional runs, while the speed of agent delivery was stepped up to 1 mL/min, we obtained similar time courses for the proportional (percentage) changes in the fluorescence emission (Figure 10; Run 2–4). However, the absolute values of the signal (expressed as the number of pixels) varied substantially. In some channels, there was a great reduction of the bacterial biomass after the pump-controlled (1 mL/min) steady injection of the agent. This may be attributed to the cleansing effect of the product. A dramatic removal of biomass happened, even though the preceding step (manual, i.e., much faster injection of fluorescent probes dissolved in saline but without surfactants) left the biofilms mostly undisturbed (Figure 11).

Despite the variability of biomass after delivering the agent to the area of a respective viewing field, graphs of fluorescence emission in the percentage of the brightest frame showed a steady increase of the PI (red) signal and a prevalent decrease of the Syto 9 (green) signal. The time course trajectories for the PI signal in all four assays followed a very similar pattern (Figure 10).

**Figure 12:**
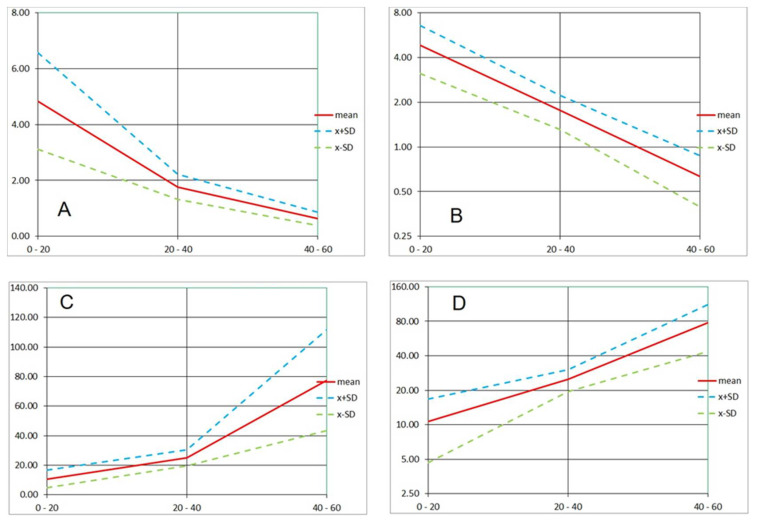
Timing of the Product “A” action. **A** and **B**: Rates of red (PI) signal change. **C** and **D**: Doubling times of red signal emission (**A** and **C**: linear scale; **B** and **D**: logarithmic scale. Mean and Standard Deviation of four runs (Run 1–4). Horizontal scale: time intervals of 60-min runs (in minutes). Vertical scales: **A** and **B**: h^−1^; **C** and **D**: minutes.

To better express the dynamics of the disinfectant impact, we calculated the rate of change of red (PI) signal and its doubling time for each run (see 3. Recommended Procedure). The data are summarized for the assays with the sample delivery at 1 mL/min together with the run having the sample delivered at 0.5 mL/min (Figure 10). In general, during the three 20-min intervals after injecting the disinfectant, the PI signal rate of change decreased exponentially (i.e., linearly in the log_2_ scale; Figure 12A,B). Consequently, the average doubling time of the PI signal intensity increased nearly exponentially over the same period (again, linearly in the log_2_ scale; Figure 12C,D; Table 2: Method 1).

**Figure 13:**
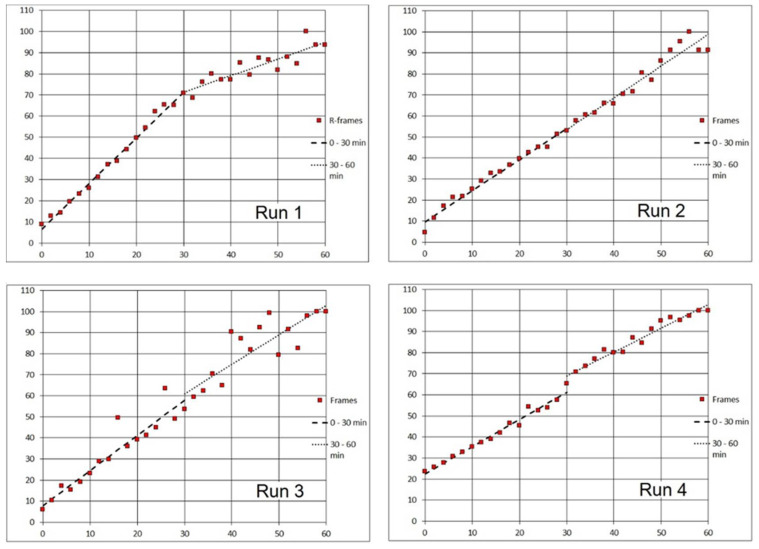
Time-lapse records of PI (red) fluorescence signal from a mixed biofilm of *S. epidermidis* and *E. coli* exposed to Product “A” (Run 1–4; same data as Figure 10). Horizontal axis: time in minutes. Vertical axis: % of maximum frame luminance. Linear best fit for two 30-min segments after the delivery of product.

With no better approximation available, the above rate of change (and doubling time) calculations had been based on single “boundary” values of luminance captured at time instants representing arbitrary time brackets (e.g., 60, 40, etc. minutes). These quantities apparently fluctuated around some central (mean) values. In order to make more accurate estimates of the rate parameters, we assessed linear trends over larger segments of time (0.5 h; Figure 13: Run 1–4; Table 2: Method 2). In all assays using either method, the average doubling time of the PI signal intensity increased with time, as the process slowed down.

### 4.2. Product “B”

**Figure 14:**
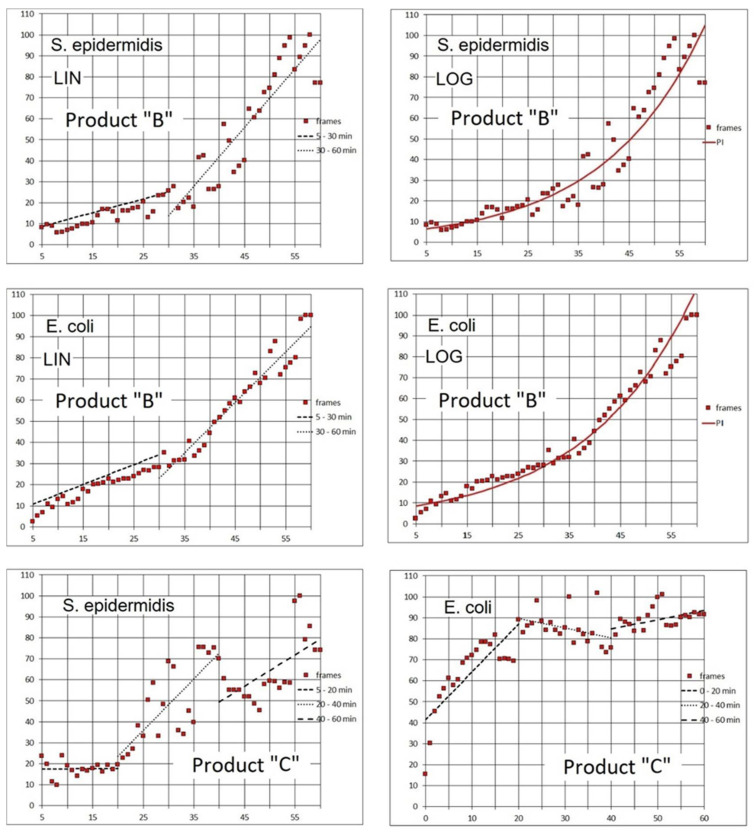
Product “B”: Time-lapse records of PI (red) fluorescence signal from a biofilm of *S. epidermidis* and *E. coli* exposed to Product “B”. Horizontal axis: time in minutes. Vertical axis: % of maximum frame luminance. LIN: Linear best fit for two intervals (25 and 30 min) after the delivery of product. LOG: Exponential best fit for the entire 1-h time series after the delivery of product. Same data set used for both LIN and LOG fit calculation.

We performed additional assays with a modification of the same product, the original liquid used for soaking DIW, i.e., Product “B”. The two available species (*Staphylococcus epidermidis* and *Escherichia coli*) were used separately for the buildup of biofilm. The time-series (59 min in 1-min intervals) started immediately after the delivery (1 mL/min) of disinfectant had stopped. However, for comparability of the 1-h records with the previous experiments, we omitted the initial 5 min to prevent the initial spikes of the red signal masking the later phases of the time course (Figure 14, Product “B”, Table 3). As opposed to the assays with Product “A”, the doubling time of the PI signal for both species remained relatively stable over the one-hour period with Product “B” (Table 3).

### 4.3. Product “C”

We used this product to compare the effect of the precise timing of agent delivery that would displace the fluid layer above the biofilm just before the time series starts (delivery option (b); see 2. Materials and Methods; same as the previous assays) against a slow continuous flow with a simultaneous record of time series from the start (delivery option (a); see 2. Materials and Methods).

The assays with the delivery (1 mL/min) option (b) were carried out with separate cultures of the two species. A one-hour recording was obtained for each species in one-minute intervals. In both recordings, we skipped the starting frames, however, we had to eliminate an additional 5 frames for *S. epidermidis* because of the initial spikes of fluorescence that would mask the later signal development. The timing of PI emission was markedly different between the two species.

*E. coli* cells apparently absorbed the PI fast and the signal rose sharply during the first 20 min after the agent delivery. On the contrary, the PI signal from *S. epidermidis* biofilm remained constantly low for the first 20 min after the initial spike. Only then, there was a fast rise (Figure 14, Product “C”; Table 4).

**Figure 15:**
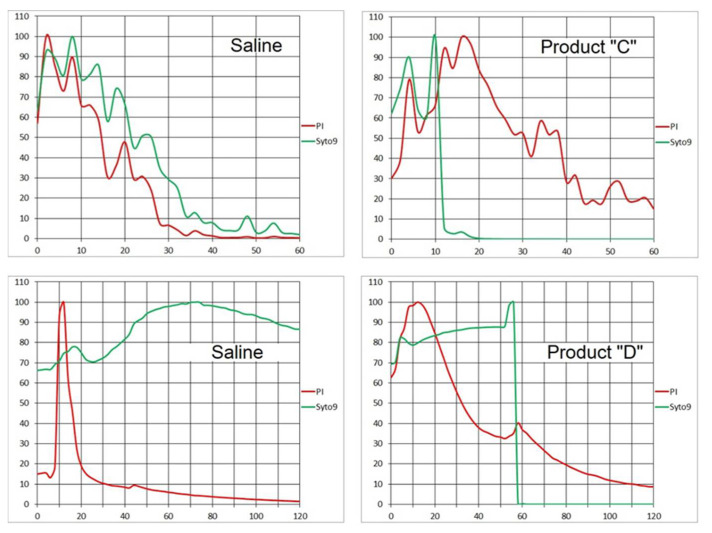
Upper diagrams (Product “C”): Time-lapse record of PI (red) and Syto 9 (green) fluorescence signal from a mixed biofilm of *S. epidermidis* and bifidobacteria exposed alternatively to 0.89% saline and the product. Time series (2-min intervals) synchronized with pump delivery (2 mL/hour). Horizontal axis: time in minutes. Vertical axis: % of maximum frame luminance. Lower diagrams (Product “D”): Time-lapse record of PI (red) and Syto 9 (green) fluorescence signal from a biofilm of *S. epidermidis* exposed alternatively to 0.89% saline and the product. Time series (2-s intervals) synchronized with pump delivery (1 mL/min). Horizontal axis: time in seconds. Vertical axis: % of maximum frame luminance.

The assay with the delivery option (a) was performed using a mixture of *S. epidermidis* and probiotic strains (see 2. Materials and Methods). The time-lapse recording started when the Linear Pump began metering the fluid from a syringe (2 mL/hour). The duration of the time series was the same as in previous assays, i.e., one hour; the recording interval was 2 min. It was expected that the agent delivery to the viewing field would take 30 min to accomplish (transfer of 1 mL; see 3. Recommended Procedure), however, the flow continued an additional 30 min till the end of the time series (Figure 15, Product “C”). A control assay was run with 0.89% saline (NaCl). For both red and green emission, it showed initial spikes within the first 10 min followed by a steady signal decay when the red emission fell below 10% in 30 min and the green emission did in 40 min. With Product “C”, after the initial spike, the green signal fell abruptly at the 14th minute. However, the red signal kept rising until the 16th minute and its decay lasted till the end of the assay when it fell to 15% at the 60th minute.

### 4.4. Product “D”

We applied the same technique (delivery option (a)) to this agent with fast bactericidal action. The assays were carried out with a clonal biofilm of *S. epidermidis.* For a detailed time resolution, snapshots were recorded in 2-s intervals during the first 2 min of the assay.

A control assay was run with 0.89% saline. The Syto 9 signal fluctuated slightly during the 2-min period with a moderately rising trend. The PI signal was slowly decreasing after a several-second initial spike. However, with the Product “D”, an instant 100% drop of the Syto 9 signal happened between 56th and 58th second of the assay while the PI signal decreased to 10% over 2 min after an initial spike (Figure 15, Product “D”).

Product “C”: Comparison of time-lapse records of PI (red) fluorescence signal between separately grown biofilms of *S. epidermidis* and *E. coli* exposed to Product “C”. Horizontal axis: time in minutes. Vertical axis: % of maximum frame luminance. Linear best fit for three 20-min segments after the delivery of product.

## 5. Discussion

The availability of diverse approaches to controlling biofilms has been growing recently. For instance, Stewart and Parker, 2019 [2] acknowledge the abundance of molecules with antibiofilm activity including novel synthetics, plant-derived agents, antimicrobial peptides, and a wide variety of nanotechnologies, photoactivated compounds and quorum-sensing inhibitors. Choosing a preferable technology depends on methods providing reliable criteria for evaluating the efficacy of a respective strategy in given circumstances. Using in vitro methods is one way of standardizing the criteria of performance. In our application, reproducible assays can be performed in terms of experimental conditions, tested organisms, fluorescent probes, and optical system settings.

Since our technique uses an indicator of bacterial plasma-membrane integrity (propidium iodide; PI), it is suitable for tracking the killing effect of membrane-active antimicrobial compounds. Although the BacLight dual-fluorescence probe had been originally designed for estimating the proportion of dead cells at the endpoint of an experiment (e.g., [12,13]), it has also been used for detecting changes to this proportion due to bactericidal agents for some time [14]. In time-lapse series, confocal stacks of the same field of view were taken at 15, 30, 45, and 60 min after chlorhexidine exposure [6]. The authors estimated the total biomass of live and dead bacterial cells using COMSTAT software. The survival rate was calculated based on the number of viable cells over time.

However, the use of fluorescent probes that intercalate in the bacterial DNA as indicators of viability is still subject to ongoing research. Generally, it is assumed that the increase of red (PI) signal is correlated with the plasma membrane permeability of the bacterial population and can be interpreted as a marker of cell death. This phenomenon may or may not be accompanied with the decay of the green (Syto 9) signal, which is partly caused by the displacement of Syto 9 molecules intercalated in the chromosomal DNA with PI molecules [15], however, it may also indicate the destruction of the chromosomal DNA. For instance, with TEM imaging, Tawakoli et al., 2013 [16] found lysis and breakdown of the biofilm cells during live/dead assays using BacLight dyes. In assays using microplate readers, Stiefel et al., 2015 [7] found that Syto 9 used without PI in the Gram-negative *Pseudomonas aeruginosa* caused the green signal to be 18 times stronger in dead cells than in live ones. In the Gram-positive *Staphylococcus aureus*, the Syto 9 emission was equal in both live and dead cells in the absence of PI. The Syto 9 signal decay (bleaching) was faster in dead cells over a period of two hours in both *P. aeruginosa* and *S. aureus*. We assumed that in our assays, at least photo-bleaching was minimized, as we used a widefield microscope that was highly light efficient and therefore preserved sample viability better than most confocal microscopes [17]. With our procedure, a time series is recorded using a low-power lens for acquiring a microscope field that represents a relatively large portion of the bottom surface of a respective flow channel. The large depth-of-field of the low-NA objective also results in signal from the entire thickness of the biofilm. Therefore, the fluorescence of colonies of bacteria is resolved rather than the emission of individual cells.

Hypothetically, an alternative approach would be to run the assays at higher magnification with maximum intensity projections (MIPs) of the CLSM stacks of biofilms. This way individual cells could be resolved for acquiring the real proportion of live and dead fraction. However, aside from greater complexity, this procedure could yield substantially different time responses of either probe emission due to a fundamentally different pattern of exposure to excitation light between the widefield and confocal microscopy.

In our study, the fluorescence of bacteria was quantified as the proportion of white pixels in individual binary frame matrices (after setting the threshold in the brightest frame of the time series, i.e., the one yielding the highest white pixel count). We used a default global thresholding algorithm of FIJI based on the probability density functions of the foreground and background [18].

The assays sampled a range of agents with different antimicrobial potency, albeit based on similar combinations of basic components. We utilized the advantage of precise agent metering for exploring their action following two approaches:

(a) Simultaneous start of metering and time-series recording
(b) Start of recording just after delivery of the bactericidal agent

In the Gram-positive *S. epidermidis*, the approach (a) helped demonstrate the almost instant decline of the Syto 9 signal when employing both fast- (Product “D”) and slow-acting (Product “C”: Figure 15) disinfectants as opposed to none or slow decay in controls. The slow-acting Product “C” is likely responsible for the difference in the absorption of PI which caused a 20-min delay in the red signal decline to 20% of the initial value compared to control. On the other hand with the fast-acting Product “D”, the PI emission decreased to 20% only one minute later than with saline (Figure 15), indicating little probe absorption difference.

While approach (a) can detect the onset of disinfectant action and is sensitive to differences among products, its quantification may become very complicated. To simplify the logic, approach (b) starts with a time point when the flow stopped, i.e., the recorded fluorescence emission is mostly independent of fluid motion. Both fluorescence signals start the time series with an initial spike. Then the Syto 9 emission appears to decline invariably and the PI signal typically rises. The rate of its increase is well defined and may be indicative of differences among bacterial strains and disinfection products. Apparently, this approach is not applicable to fast-acting agents (e.g., Product “D”) unless they are diluted. Otherwise, all cells would be dead before the time series starts.

The agents tested in our assays had more than one way to impact the in vitro biofilm microbiome. The detachment of bacterial cells from the surface being disinfected was an additional major effect. Depending on a particular species or strain, this complicates the testing method based on direct observation of changes at the cellular or micro-colony level. Using approach (b), the delivery of “agents” to the viewing frame had been calibrated so that BacLight probes were not washed out excessively. Still, there was some instability around the start of the time series due to spikes of fluorescence signal and residual motion of the channel contents. These initial spikes of fluorescence signal were generally observed in all series following both approaches. If taken into account, all other values of the sequence would become mostly negligible and the proportional dynamics of the signal would have low resolution. Therefore, we regarded them as outliers and excluded them from the datasets. The remaining values were then recalculated as percentages of the pixel count in the brightest frame for easier visual inspection of graphs.

Specifically, in Runs 1–4 (Product “A”; Figure 10) the zero time (origin) was set at 2 min; for the Product “B” (Figure 14), the zero was 5 min. Without the above corrections (subtraction of the frames that captured the start) the initial changes in the viewing field translated differently in the two emission channels (Table 5). For the PI emission, an initial “flush” led to a dramatic decrease in the signal. On the contrary, the Syto 9 emission was only diminished in Run 4, while in Runs 2 and 3, there was a slight increase. Run 1 was anomalous in that the “flush” fortuitously brought a major cluster of micro-colonies into the viewing field which then remained in place for the rest of the assay (1 h; Figure 6). We can assume that the PI signal in Runs 2 to 4 and the Syto 9 signal in Run 4 spiked before the flush so that rinsing out the biomass caused a major drop in brightness. On the contrary, a delayed spike of Syto 9 signal in Runs 2 and 3 would compensate for the loss of biomass, making the brightness quasi-constant.

For slower acting agents such as Products “A” and “B”, designed for disinfecting rough-surface materials without damaging their texture, the bactericidal effect was demonstrated by independent test procedures. Based on PI dynamics, the 10 times increase in the emission (that could be attributed to a log reduction of live biomass) happened in tens of minutes. Inspecting PI signal increase in Figure 10, we can approximate the log reduction of living biomass as 55 min in Run 1, less than 30 min in Run 2, 15–25 min in Run 3, and more than 60 min in Run 4. However, using the rate of increase or signal doubling time (Figure 12; Table 2, Table 3 and Table 4) appears to better characterize the fluorescence signal dynamics.

Variation of the white pixel proportion with time allowed us to compute the rate of change and the doubling time (or decay time) of the total red or green signal emitted by the biofilm. This conversion of captured fluorescence values to rates makes the evaluation of cellular-level changes independent of cell density, thus sufficiently robust even when a substantial part of the observed population is removed by the agent. Along these lines, it is also worth noting that the rate of change over a certain longer interval equals the average of the rates over short equal segments of that interval.

Using approach (b), the highest value of the PI (red) fluorescence emission (100%) was typically found near the end of the time series. Thus, we were assessing the portion of the final damage achieved at particular points of the sequence. As we chose the length of the assay (e.g., 60 min), we estimated the timing of proportional damage relative to the arbitrary final outcome. It is unclear if the PI emission kept increasing after the time series stopped. The information is also missing on whether all or only a fraction of the population was harmed. On the other hand, the shape of the emission course indicates either its continuation when still increasing at the end (positive rate of change or doubling time) or stagnation when a plateau is reached (rate of change near zero).

The results of assays with the simultaneous onset of metering the fluid and the time-lapse record, i.e., approach (a), may reflect a rinsing effect by the steady influx of fresh solvent (saline) above the biofilm, constantly eluting fluorescent probes from the biofilm’s exopolysaccharide matrix (EPS) and the surface of bacterial cells. The probes had been partly removed before they could be absorbed by the cells. When bactericidal agent (“C”) was brought instead of the “solvent”, the cells were made permeable and, possibly, the structure of EPS was altered. Consequently, the biofilm absorbed more PI which caused longer-lasting red emission. At the same time, the Syto 9 signal was quenched faster than in control, as the DNA was being broken down.

It remains to be explained, why the fluorescent events happened much earlier than one millilitre of fluid would have been injected, since the leading edge of the agent arrival was expected about 30 min after start according to the “fast flow” calibration (see 3. Recommended Procedure). Given the physical dimension of the Ibidi channel profile (height 0.4 mm; width 3.8 mm), slow flow velocity probably caused major deviation from a laminar (plug-flow) pattern.

## 6. Conclusions

We presented here a novel technique of using a commercially available microfluidic device and time-lapse fluorescence microscopy to study the effects of disinfectants on bacterial biofilms. This method can be used to bridge the gap between bulk measurements such as MBEC [3] and the detailed investigations of specific phenomena at the cellular level (e.g., [6]). When engaging additional fluorescent probes, such as fluorescently labelled dextrans [6,19], the disinfectant impact can be traced to the extracellular matrix and its function in biofilm survival. The procedure is apparently capable of detecting the instant vs. delayed effect of an agent and the differences between different types of microbes (e.g., Gram-positive vs. Gram-negative; Figure 14). Not only is that helpful for comparing different brands of disinfectants, but it may also become a guide for making targeted modifications while developing new products.

From a practical viewpoint, the test described here is relatively fast, and when used with a well-defined biofilm, it has the potential for high reproducibility. In addition to capturing the course of damage to bacterial cells, it can help quantify the biofilm detachment (sloughing) caused by a tested agent. Since it is based on the dynamics of nucleic acid response, this approach may also be considered for testing antifungal, anti-protozoan or even antiviral products. However, before making conclusions about a particular disinfectant taken to the test, the results must be validated by comparison with alternative methods.

## Figures and Tables

**Table 1:** Timing of cell suspension arrival in the central part of Ibidi flow cell channel (in seconds from the start of pumping).

*E. coli* Suspension Sample	Arrival at Viewing Field (seconds)
A	55
B	50
C	50
D	45
Mean	50
SD	2.9

**Table 2:** Estimates of the signal doubling time of PI (red) fluorescence emission in four assays of Product “A”.

Method 1		Method 2	
Interval	Doubling Time (min)	Interval	Doubling Time (min)
0–20 min	10.7	0–30 min	
20–40 min	24.9	Run 1	8.7
40–60 min	77.6	Run 2	12.1
		Run 3	10.4
		Run 4	20.7
		30–60 min	
		Run 1	71.9
		Run 2	33.8
		Run 3	39.7
		Run 4	51.9

NOTE: Time scale was shifted to the right by 2 min to eliminate the “outlier” effect of initial spikes.

**Table 3:** Estimates of the signal doubling time of PI (red) fluorescence emission in two assays of Product “B”.

	Linear Estimate	Exponential Estimate
Interval	Doubling Time (min)	Doubling Time (min)
*S. epidermidis*		
5–30 min	16.5	
		13.7
30–60 min	10.7	
*E. coli*		
5–30 min	15.0	
		14.7
30–60 min	14.7	

NOTE: Time scale was shifted to the right by 1 min to eliminate the “outlier” effect of initial spikes.

**Table 4:** Estimates of the signal doubling time of PI (red) fluorescence emission in two assays of Product “C”.

*S. epidermidis*		*E. coli*	
Interval	Doubling Time (min)	Interval	Doubling Time (min)
5–20 min	–53.6	0–20 min	7.9
20–40 min	10.8	20–40 min	−85.9
40–60 min	241.1	40–60 min	73.1

NOTE: Time scale was shifted to the right by 1 min to eliminate the “outlier” effect of initial spikes.

**Table 5:** The relative values of Syto 9 (green) and PI (red) fluorescence emission during the first 2 min of the four assays of Product “A”.

Syto 9 Emission			PI Emission		
Assay	0 min	2 min	Assay	0 min	2 min
Run 1	0.07	23.29	Run 1	2.16	26.42
Run 2	9.04	10.71	Run 2	126.42	13.03
Run 3	14.47	16.74	Run 3	110.20	9.00
Run 4	77.00	0.59	Run 4	37.15	0.29

NOTE: Starting time (0 min) equals the stop time of metering pump.

## Supplementary Materials

The following are available online at https://www.mdpi.com/2076-2607/8/11/1837/s1, Figure S1: The Ibidi flow cell (Slide VI^0.4^ Ibi Treat; Ibidi, USA) mounted firmly in the microscope stage insert (Universal Insert 160x110 mm, Applied Scientific Instruments, USA).
