# Supplementary material for: Evaluating the Effects of Disinfectants on Bacterial Biofilms Using a Microfluidics Flow Cell and Time-Lapse Fluorescence Microscopy
Source: Microorganisms. 2020 Nov 22;8(11):1837. doi: 10.3390/microorganisms8111837 (PMC7700140; doi:10.3390/microorganisms8111837)
Supplement: Supplementary file 1 [file microorganisms-08-01837-s001.pdf]

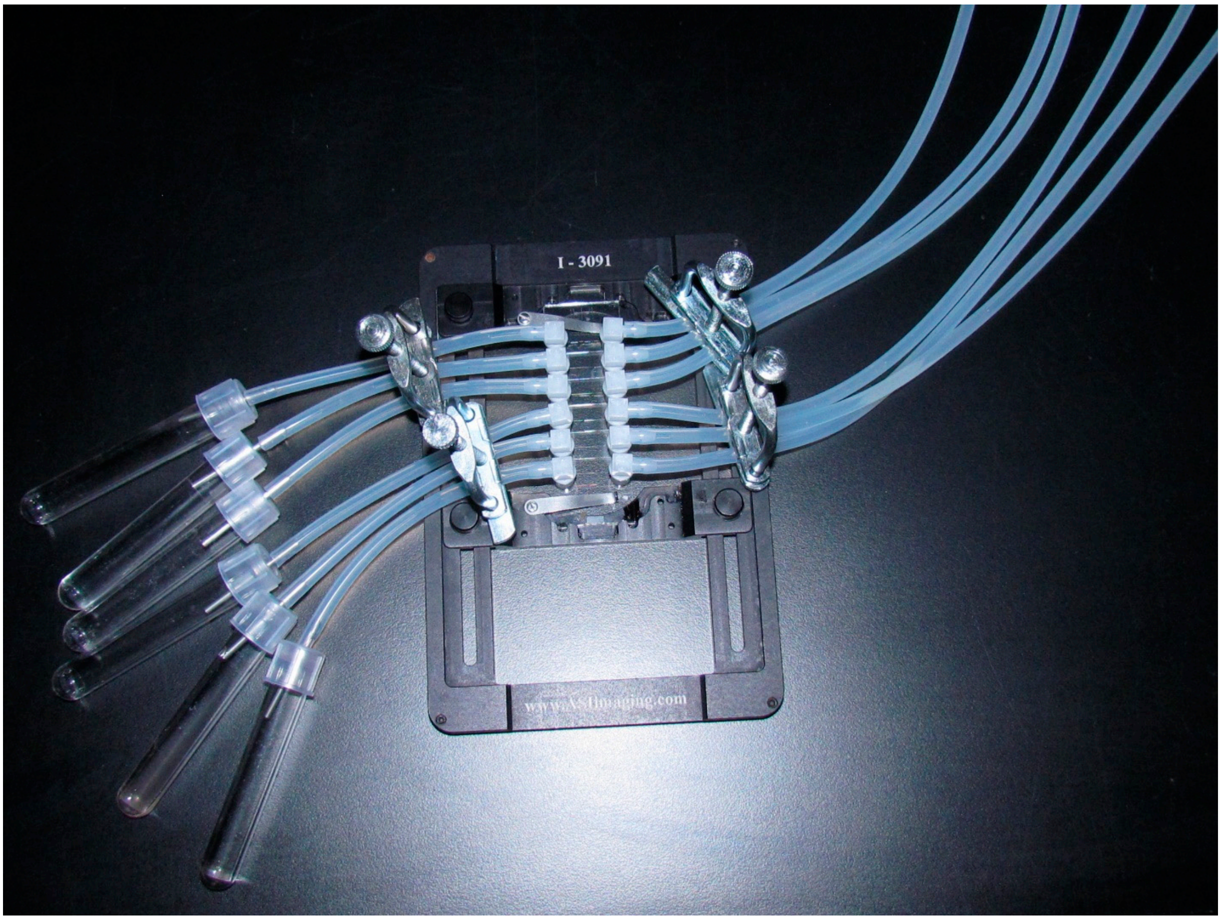

Supplementary Figure 1. The Ibidi flow cell (Slide VI$^{0.4}$ Ibi Treat; Ibidi, USA) mounted firmly in the microscope stage insert (Universal Insert 160x110 mm, Applied Scientific Instruments, USA). Except for the six replaceable syringes attached to the inflow tubing (on the right; not shown), this picture covers the entire assembly. Both inflow and outflow tubing are attached to the flow cell via elbow plug connectors. On the left side, there are six waste collectors (BD-tubes) with blunt needle pieces penetrating their caps. By unplugging from their caps, the waste collectors can be replaced. During incubation, all inflow and outflow connections are clamped, as the biofilm grows under static (batch) conditions.
